# Using shared clinical decision support to reduce adverse drug events and improve patient safety

**DOI:** 10.3389/fdgth.2025.1703141

**Published:** 2025-12-04

**Authors:** Jiancheng Ye, Sophie Bronstein

**Affiliations:** Weill Cornell Medicine, Cornell University, New York, NY, United States

**Keywords:** adverse drug events, human factors, clinical decision support, health information technology, shared decision-making, patient safety, risk assessment, digital health services

## Abstract

**Background:**

Medications, while essential therapeutic tools in modern healthcare, carry the inherent risk of causing adverse drug events (ADEs) that can result in significant morbidity, mortality, and healthcare costs. Despite substantial research efforts in this domain, the majority of ADEs remain undetected due to reliance on voluntary reporting systems and inadequate surveillance mechanisms. Consequently, the true scope and impact of ADEs are likely far greater than currently recognized.

**Objective:**

To examine the role of shared clinical decision support (SCDS) in reducing adverse drug events and enhancing patient safety outcomes through systematic integration of clinical decision support systems with shared decision-making frameworks.

**Methods:**

We conducted a narrative review of literature published up to June 2025, utilizing validated patient safety frameworks to identify contextual factors, systemic challenges, and evidence-based strategies that influence adverse drug event occurrence and prevention.

**Results:**

Multiple interconnected factors contribute to ADE susceptibility, including healthcare provider competencies (inadequate monitoring, symptom recognition failures), clinical environment characteristics (technology workarounds, equipment complexity), pharmacy system factors (high-risk medication storage, limited pharmacist involvement), and patient-specific variables (polypharmacy, multimorbidity, age-related physiological changes). Critical risk determinants include provider fatigue and burnout, inadequate monitoring protocols, medication administration errors, and systemic communication failures. Successful implementations require multifaceted approaches integrating health information technology components, stakeholder engagement, customized clinical decision rules, and continuous quality improvement processes.

**Conclusions:**

Shared clinical decision support represents a paradigm shift toward patient empowerment, enabling active patient participation in healthcare decisions while leveraging technology-enhanced clinical guidance. The most promising approach to ADE elimination involves a comprehensive integration of educational initiatives, human factors engineering, robust shared clinical decision support systems, and multidisciplinary collaborative care models.

## Introduction

Medications constitute indispensable therapeutic tools in contemporary healthcare, serving as primary interventions for treating diverse pathological conditions, managing symptomatic presentations, and optimizing patient outcomes across various clinical settings (1). However, the pharmaceutical landscape presents a complex duality, as these same therapeutic agents can precipitate adverse drug events (ADEs) ranging from minor discomfort to life-threatening complications (2). The intricate nature of modern drug therapy, particularly in patients with multiple comorbidities and complex medication regimens, exponentially increases ADE susceptibility. Addressing this critical safety challenge represents a fundamental priority in advancing patient safety standards and healthcare quality metrics.

Adverse drug events (ADEs) are broadly defined as injuries resulting from medical interventions related to drugs, encompassing medication errors, adverse drug reactions, allergic responses, and overdose incidents (3). This broad definition captures the multifaceted nature of drug-related harm and acknowledges both preventable and non-preventable adverse outcomes. ADEs impose a significant burden on healthcare systems worldwide, with wide-ranging implications for patient safety, clinical outcomes, and resource utilization. In the U.S., ADEs contribute to approximately 2 million hospital admissions annually, resulting in extended lengths of stay and substantially increased healthcare expenditures (4). Additionally, ADEs account for approximately 1 million emergency department visits and 3.5 million physician office consultations each year, representing a significant strain on healthcare resources and capacity (3, 5). Certain demographic groups demonstrate heightened susceptibility to ADEs, with elderly populations representing a particularly vulnerable cohort (6). Research indicates that ADE prevalence ranges from 4.8% to 37% among elderly patients with cognitive disorders, reflecting the complex interplay between aging physiology, polypharmacy, and comorbid conditions (7). A study evaluating ADEs through combined patient self-reporting and clinical review found that 78% of individuals over 70 years of age had experienced at least one ADE within a six-month period, highlighting the pervasive nature of this safety concern in geriatric populations (8).

Despite these alarming statistics, the true prevalence of ADEs likely represents a significant underestimation due to systematic underreporting and limitations in current detection methodologies. Healthcare systems predominantly rely on voluntary reporting mechanisms and error tracking systems, which capture only a fraction of actual events. Conservative estimates suggest that merely 10%–20% of medication errors are formally reported, indicating that the actual scope of ADEs may be five to ten times greater than currently documented (9). The consequences of ADEs extend far beyond immediate clinical presentations, encompassing significant morbidity and mortality outcomes. Beyond the direct health impacts, ADEs precipitate increased healthcare utilization patterns, elevated treatment costs, and diminished functional status for affected patients (7). The economic burden includes direct costs associated with additional treatments, extended hospitalizations, and emergency interventions, as well as indirect costs related to lost productivity and reduced quality of life.

The severity of harm associated with ADEs exists along a continuum, ranging from minimal or no detectable harm to severe morbidity and mortality. Research examining over 1,000 prescribing errors revealed that 11.5% were likely to have caused patient harm, while 19.3% required additional monitoring interventions (10). This spectrum includes “near miss” events that, while not causing immediate harm, possess the potential for significant adverse outcomes if left unaddressed. These near-miss events are estimated to occur in 5%–10% of hospitalized patients but remain substantially understudied in current literature (11).

Table 1 demonstrates the classification and characteristics of adverse drug events. ADEs can be systematically classified into two primary categories: Type A (augmented) and Type B (bizarre) reactions (12). Type A reactions are dose-dependent and predictable, frequently resulting from medication errors such as incorrect dosing, inappropriate frequency, or excessive duration of therapy. These errors are largely preventable through implementation of proper prescribing protocols and enhanced monitoring systems (13). Type A reactions are commonly associated with frequently prescribed medications and often involve prescribing errors, making their reduction a critical clinical priority (14). Type B reactions are unpredictable and dose-independent, typically involving allergic or idiosyncratic responses. While these reactions are more challenging to prevent due to their unpredictable nature, proper patient history taking, allergy documentation, and genetic testing (where applicable) can reduce their incidence (15).

**Table 1:** Classification and characteristics of adverse drug events.

ADE type	Characteristics	Predictability	Prevention potential	Common examples
Type A (Augmented) (12)	Dose-dependent, predictable	High	High (>80%)	Bleeding from anticoagulants, sedation from benzodiazepines
Type B (Bizarre) (13)	Dose-independent, unpredictable	Low	Low (<20%)	Anaphylaxis, idiosyncratic reactions
Near Miss Events (14)	Potential for harm, no immediate damage	Variable	High	Incorrect dosing caught before administration
Prescribing Errors (15)	Wrong drug, dose, or patient	High	Very High (>90%)	Inappropriate medication selection

Prescribing errors represent a significant contributor to Type A ADEs, occurring at multiple stages of the medication-use process, including prescribing, transcription, dispensing, and administration phases. Common prescribing errors include inappropriate medication selection, incorrect dosing calculations, and failure to consider patient-specific factors such as renal or hepatic function, age-related physiological changes, and concurrent medications. A study found that nearly 94% of hospitalized patients experienced at least one prescribing error during their admission, underscoring the pervasive nature of this problem (16). Medication administration errors constitute another major cause of ADEs, occurring when incorrect medications are administered, correct medications are given in wrong doses or via inappropriate routes, or medications are administered at incorrect times (17). Contributing factors include clinical interruptions during medication preparation and administration, similar packaging or labeling of different pharmaceutical products, and increasingly complex medication regimens that challenge healthcare providers' cognitive capacity and attention to detail (18).

## Methods

### Search strategy

We conducted systematic searches of PubMed, EMBASE, CINAHL, Cochrane Library, and Web of Science databases for literature published up to June 2025. Search terms included combinations of: “adverse drug events,” “medication errors,” “clinical decision support,” “shared decision-making,” “health information technology,” “CPOE,” “patient safety,” “prescribing errors,” and “medication reconciliation.” Boolean operators (AND, OR) were used to combine search terms. We supplemented database searches with manual review of reference lists from key articles and citation tracking of seminal publications in the field.

### Inclusion and exclusion criteria

We included peer-reviewed research articles, systematic reviews, meta-analyses, and clinical guidelines published in English that addressed: (1) epidemiology and risk factors for adverse drug events; (2) health information technology interventions for ADE prevention; (3) clinical decision support system implementation and effectiveness; (4) shared decision-making frameworks and patient engagement strategies; (5) geriatric-specific medication safety concerns; and (6) multidisciplinary approaches to medication safety. We excluded conference abstracts, editorials without original data, and studies focusing exclusively on specific therapeutic areas (e.g., oncology) without broader applicability to general ADE prevention.

### Synthesis approach

We utilized the World Health Organization's Conceptual Framework for the International Classification for Patient Safety (19) as an organizing structure to categorize findings and identify key themes related to ADE occurrence and prevention. Data extraction focused on: (1) risk factors contributing to ADEs across patient, provider, and system levels; (2) types and characteristics of SCDS interventions; (3) implementation strategies and barriers; (4) reported clinical and safety outcomes; and (5) economic implications. We synthesized findings narratively, organizing evidence by thematic areas including risk assessment, intervention strategies, implementation frameworks, and outcomes evaluation. Conceptual frameworks were developed to integrate evidence across studies and illustrate relationships between risk factors, interventions, and outcomes.

## Shared clinical decision support

Shared Clinical Decision Support (SCDS) represents an innovative healthcare delivery model that integrates clinical decision support systems (CDSS) with shared decision-making (SDM) frameworks to enhance patient care quality and safety outcomes. This approach embodies a collaborative paradigm where healthcare professionals and patients work synergistically to make clinical decisions that align with patient values, preferences, and lifestyle considerations while incorporating evidence-based clinical guidelines and best practices. The fusion of CDSS capabilities with SDM principles creates a powerful framework for addressing complex clinical scenarios, particularly in medication management where the risk of ADEs is substantial (20). This integration leverages health information technology to provide real-time, evidence-based recommendations while ensuring that patient perspectives and preferences remain central to the decision-making process.

### The role of health information technology in SCDS

Health Information Technology (HIT) is a cornerstone of SCDS, providing the necessary tools and infrastructure to support both clinicians and patients in making informed decisions. Table 2 illustrates the key components of health information technology in SCDS.

**Table 2:** Key components of health information technology in SCDS.

HIT component	Primary function	ADE prevention mechanism	Implementation challenges
Electronic Health Records (EHRs) (21)	Patient data management	Real-time allergy and interaction checking	Data quality, interoperability
Computerized Physician Order Entry (CPOE) (22)	Direct order entry by providers	Elimination of transcription errors	User interface design, workflow integration
Clinical Decision Support Systems (CDSS) (23)	Evidence-based recommendations	Automated risk assessment and alerts	Alert fatigue, rule maintenance
Patient Portals (24)	Patient engagement platform	Enhanced communication and medication reconciliation	Digital literacy, access barriers
Secure Messaging (25)	Provider-patient communication	Clarification of medication instructions	Response time management

#### Electronic health records (EHRs): the foundation of digital healthcare

EHRs serve as digital repositories containing complete patient information, including medical histories, current medications, documented allergies, laboratory results, imaging studies, and clinical notes (26). EHRs facilitate seamless information sharing among healthcare providers, ensuring continuity of care and reducing the risk of errors attributable to incomplete or inaccurate information (27, 28). The integration of EHRs with CDSS enables real-time alerts and clinical reminders about potential drug interactions, allergy contraindications, and other critical clinical considerations at the point of care (21).

#### Computerized physician order entry (CPOE): streamlining prescription processes

CPOE systems enable healthcare providers to enter medication orders and clinical instructions directly into computerized systems, significantly reducing prescribing errors by eliminating handwriting legibility issues, transcription errors, and incomplete order problems (29). When integrated with CDSS capabilities, CPOE systems provide immediate feedback regarding potential drug interactions, dosage errors, and clinical contraindications, creating a robust safety net that reduces ADE risk at the point of prescribing (30).

#### Clinical decision support systems (CDSS): intelligent clinical guidance

CDSS represent sophisticated software applications designed to assist healthcare providers in clinical decision-making processes. These systems analyze patient data from EHRs and other sources to generate evidence-based recommendations, alerts, and clinical reminders (31). Advanced CDSS capabilities include allergy alerts when contraindicated medications are ordered, drug interaction warnings when potentially harmful combinations are prescribed, and alternative medication suggestions based on patient-specific factors such as age, weight, renal function, and hepatic status (23).

#### Patient portals: empowering patient engagement

Patient portals are secure, web-based platforms that enable patients to access their health information, communicate with healthcare providers, and actively participate in their care management (24). Through these platforms, patients can review their medication lists, access laboratory results, review visit summaries, and receive educational materials and decision aids (32, 33). Patient portals facilitate shared decision-making by providing patients with relevant clinical information and enabling active participation in treatment discussions and decisions (33).

#### Secure messaging systems

Secure messaging platforms allow patients and healthcare providers to communicate electronically while maintaining privacy and security standards (25). These systems can be utilized to address medication-related questions, clarify treatment instructions, and provide follow-up care guidance (34). Secure messaging enhances SCDS implementation by ensuring that patients maintain direct communication access with their healthcare teams, helping prevent misunderstandings and medication-related errors (35).

### Implementing shared clinical decision support in clinical practice

Successful SCDS implementation requires planning, multi-stakeholder collaboration, and continuous evaluation processes. The implementation framework should address technical requirements, workflow integration, user training, and performance monitoring to ensure optimal outcomes. Table 3 demonstrates the SCDS implementation framework.

**Table 3:** SCDS implementation framework.

Implementation phase	Key activities	Evaluation metrics
Assessment and Planning (36)	Needs analysis, stakeholder engagement	Baseline ADE rates, user requirements
System Integration (37)	HIT system integration, workflow design	System interoperability, user acceptance
Training and Education (38)	Staff training, patient education	Competency assessments, usage rates
Pilot Implementation (39)	Limited rollout, performance monitoring	ADE reduction, user satisfaction
Full Deployment (40)	System-wide implementation	Clinical outcomes, cost-effectiveness
Continuous Improvement (41)	Ongoing optimization, updates	Sustained performance, innovation adoption

#### Identifying priority clinical areas

The initial step in SCDS implementation involves identifying clinical areas where the intervention can achieve maximum impact (42). This process typically involves analyzing ADE surveillance data to identify high-risk medications, vulnerable patient populations, and clinical settings with elevated risk profiles. Implementing SCDS in chronic disease management, such as diabetes, hypertension, and cardiovascular disease, where medication regimens are complex and ADE risk is elevated, often provides substantial benefits.

#### Stakeholder engagement strategies

Successful SCDS implementation requires engagement of all stakeholders, including healthcare providers, patients, pharmacists, information technology professionals, and administrative leadership (36). Stakeholder engagement should begin early in the implementation process to ensure that diverse perspectives and requirements are incorporated into system design and deployment strategies (43). This engagement can be facilitated through focus groups, stakeholder surveys, and regular collaborative meetings (36).

#### System integration requirements

SCDS must be seamlessly integrated with existing health IT infrastructure, including EHRs and CPOE systems, to ensure that clinical decision support is readily available during routine clinical workflows. Integration involves ensuring smooth data flow between systems and making CDSS alerts and recommendations easily accessible to healthcare providers during patient care activities (37).

#### CDSS rule development and customization

The effectiveness of SCDS depends substantially on the quality and clinical relevance of CDSS rules and algorithms (44). These rules should be based on current clinical guidelines and evidence-based practices while being customized to meet the specific needs of the clinical environment and patient population (44). For example, CDSS rules for anticoagulation management should incorporate patient-specific factors such as age, renal function, and potential drug interactions to provide optimal safety and efficacy recommendations (45).

#### Education and training programs

Education and training programs are essential components of SCDS implementation (38). Healthcare providers require training on effective utilization of SCDS tools and integration of shared decision-making principles into clinical practice. Training curricula should address CDSS alert interpretation, patient portal utilization for shared decision-making, and effective patient engagement strategies in treatment discussions (46).

#### Performance evaluation and continuous improvement

Ongoing evaluation is crucial for ensuring that SCDS achieves its intended objectives and for identifying opportunities for system optimization. Evaluation methodologies include ADE rate analysis, healthcare provider and patient satisfaction surveys, and system usability assessments (47). Based on evaluation findings, SCDS can be continuously refined to address identified issues and optimize effectiveness.

## Barriers and challenges

### Systemic contributors to adverse drug events

The healthcare environment contains numerous interconnected factors that contribute to ADE occurrence, many of which are not unique to specific patient populations. Understanding these systemic challenges is essential for developing effective prevention strategies. Table 4 presents the risk factors for adverse drug events.

**Table 4:** Risk factors for adverse drug events by category.

Risk category	Specific factors	Impact level	Prevention strategies
Healthcare Provider Factors			
Insufficient monitoring (48)	Inadequate hemodynamic, telemetry, or lab monitoring	High	Enhanced monitoring protocols, automated alerts
Symptom recognition failure (44)	Inability to identify drug-induced symptoms	High	Education, decision support tools
Prescribing cascade (49)	Adding medications for drug-induced symptoms	Medium–High	Medication review, deprescribing protocols
Clinical Environment Factors			
IV tubing compatibility (50)	Wrong route administration errors	Medium	Standardized tubing, color coding
Multiple IV pump systems (51)	Programming errors, dose confusion	Medium	Standardized equipment, training
Technology workarounds (52)	Bypassing safety features	High	Improved interface design, workflow integration
Physical environment (53)	Falls risk increasing bleeding complications	Medium–High	Environmental modifications, risk assessment
Pharmacy Factors			
High-risk medication storage (54)	Concentrated solutions in patient areas	Very High	Centralized storage, restricted access
Lack of central pharmacy (55)	Nurses performing mixing/dispensing	High	Centralized pharmacy services
Limited pharmacist involvement (56)	No participation in rounds or prescription review	High	Integrated clinical pharmacy services
Patient Factors			
Action errors (57)	Inappropriate medication behaviors	Medium	Patient education, behavioral interventions
Mental errors (11)	Misunderstanding medication management	Medium	Clear communication, health literacy support

**Figure 1:**
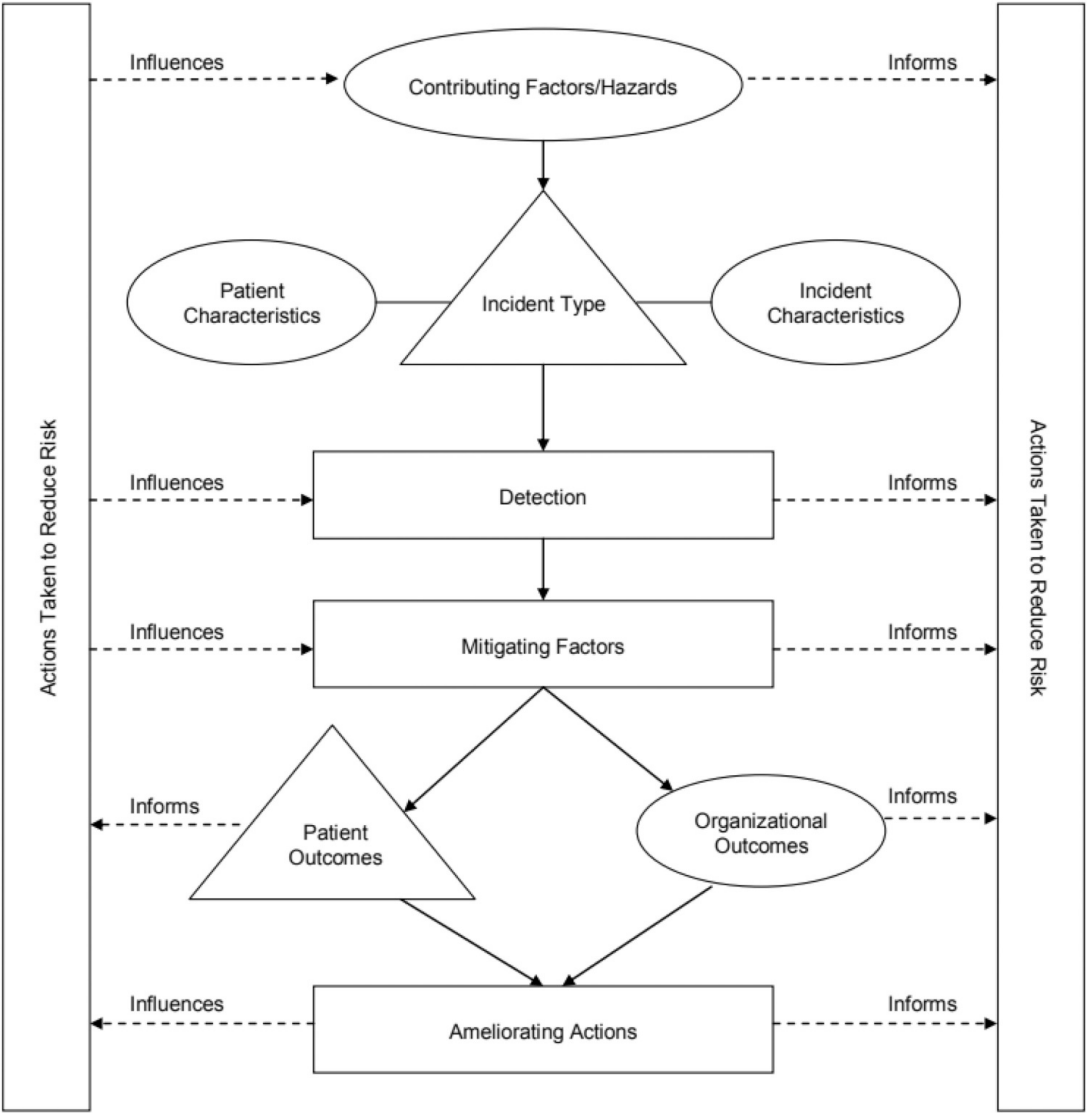
The conceptual framework for the international classification of patient safety (19).

We applied the Conceptual Framework for the International Classification for Patient Safety (Figure 1) that was developed by the World Health Organization (19) to understand the risk factors of ADE. This framework was designed to enable categorization of patient safety information using standardized sets of concepts with agreed definitions, preferred terms and the relationships between them being based on an explicit domain ontology (e.g., ADE). The framework has been be a genuine convergence of international perceptions of the main issues related to patient safety and has facilitated the description, comparison, measurement, monitoring, analysis and interpretation of information to improve patient care (19).

#### Healthcare provider-related factors

Healthcare provider factors primarily encompass insufficient clinical monitoring and inadequate recognition of medication-related symptoms. Insufficient monitoring—whether involving hemodynamic parameters, cardiac telemetry, or laboratory values—can affect both physicians and nursing staff, potentially leading to ADEs (58). Research indicates that inadequate monitoring contributes to 17.6% of preventable ADEs, highlighting the critical importance of systematic surveillance protocols (48). The inability to recognize symptoms potentially related to medication use represents another significant risk factor. When healthcare providers cannot identify symptoms that may indicate an ADE, this creates a dangerous cycle involving continued medication use, symptom progression, and increased patient harm. Often, the failure to distinguish drug-induced symptoms from underlying medical conditions leads to the addition of additional medications, a phenomenon known as the “prescribing cascade,” which increases the risk of drug-drug interactions and additional ADEs (49).

#### Clinical environment challenges

The clinical environment itself can significantly increase patient ADE risk. Universal compatibility of intravenous therapy tubing represents a common source of medication errors, typically involving wrong-route administration (50). The utilization of multiple different IV pump systems with varying programming requirements for different medications can also increase ADE risk through programming errors and dose confusion. Complex or cumbersome technology at the human-machine interface may lead providers or nurses to develop workarounds for frustrating obstacles, which can ultimately result in medication errors and ADEs (52). Additionally, the physical environment can place frail patients at increased risk for falls, which subsequently increases the risk of bleeding complications from anticoagulant medications or antiplatelet agents (53).

#### Pharmacy system factors

Certain pharmacy-related factors are recognized as significant ADE risk contributors. The storage of high-risk medications, such as concentrated potassium solutions, in patient care areas has demonstrated increased ADE risk (11). The absence of a centralized pharmacy capable of providing ready-to-use medication units increases ADE risk by requiring nursing staff to perform medication mixing and dispensing activities, introducing additional opportunities for errors.

Some clinical settings and units lack pharmacist participation in clinical rounds when medications are typically prescribed and when prescribing errors are most likely to occur. Some healthcare facilities utilize pharmacists only to review high-risk medication prescriptions rather than implementing prescription verification processes. While this approach may appear cost-effective, it creates the risk of missing critical errors with traditionally lower-risk but frequently prescribed medications.

#### Patient-Related factors

Patients themselves can contribute to increased ADE risk in both inpatient and outpatient settings. Research demonstrates that 25% of ADEs within a large health maintenance organization were attributed to patient errors. These patient errors can be categorized as action errors (inappropriate patient behaviors) or mental errors (errors involving patient thought processes) (57). Action errors might include patients consuming alcohol while taking contraindicated medications despite receiving appropriate counseling. Mental errors might involve patients continuing to take their home medications while hospitalized, mistakenly assuming they are responsible for their own medication management during hospitalization and that continuing all home medications is safe (11).

### Geriatric-specific risk factors for adverse drug events

While the aforementioned risk factors affect all patient populations, geriatric patients face additional, population-specific risks that significantly increase their ADE susceptibility compared to younger adults (13). The association between advanced age and increased ADE rates stems from multiple interconnected factors, including increased comorbidity burden, polypharmacy, physiological changes, and functional decline or frailty. Table 5 demonstrates the geriatric-specific risk factors for adverse drug events.

**Table 5:** Geriatric-specific risk factors for adverse drug events.

Risk factor	Mechanism	Clinical impact	Mitigation strategies
Multimorbidity			
Multiple chronic conditions (59)	Contraindications, drug interactions	High	Comprehensive medication review
Polypharmacy			
≥5 medications regularly (60)	Drug-drug interactions, adherence issues	Very high	Deprescribing protocols, medication reconciliation
2–6 prescribed medications (61)	Complexity management	High	Simplified regimens
1–3.4 non-prescribed medications (13)	Unrecognized interactions	Medium–High	Comprehensive medication history
Physiological Changes			
Renal function decline (14)	Altered drug clearance	High	Renal dose adjustment protocols
Hepatic function changes (62)	Altered drug metabolism	Medium–High	Hepatic dosing considerations
Body composition changes (63)	Altered drug distribution	Medium	Individualized dosing strategies
Increased drug sensitivity (64)	Enhanced pharmacological effects	High	Lower starting doses, careful titration
Functional Changes (Frailty)			
Diminished strength/endurance (65)	Fall risk, medication handling	High	Environmental modifications, assistance
Reduced physiologic reserve (66)	Decreased ability to handle stressors	High	Conservative treatment approaches
ADL dependency (67)	Medication management difficulties	Medium–High	Support services, simplified regimens

#### Multimorbidity and comorbidity impact

As patients age, accumulating comorbidities significantly increase ADE risk. Geriatric patients represent a population with higher disease burden and more complex health conditions that may create relative or absolute contraindications to specific medications (68). The prevalence of this risk factor is remarkable: systematic reviews indicate that 95.1% of patients over 65 years in primary care settings have multimorbidity (69). Research by Jose et al. identified multiple disease states as one of the two most common predisposing factors for ADEs in hospital settings (70).

#### Polypharmacy complications

Polypharmacy, defined most commonly as the regular use of five or more medications, represents the other most common predisposing factor for ADEs in geriatric populations. Older adults face the greatest risk for polypharmacy, as medication lists typically expand with advancing age, leading to increased potential for medication interactions and adverse effects when new medications are prescribed. Research indicates that patients over 65 years use an average of two to six prescribed medications, in addition to one to 3.4 non-prescribed medications, which are often not identified or considered in clinical settings (13). Multiple studies have established links between polypharmacy and adverse events in geriatric patients (13). Kojima et al. demonstrated that geriatric patients taking five to eight medications had significantly increased risk for ADE-related hospitalizations and fall-related injuries (71).

#### Physiological changes and pharmacological implications

Age-related physiological changes represent critical factors in geriatric ADE risk when not appropriately considered in prescribing decisions. These changes include alterations in pharmacokinetics and pharmacodynamics, increased sensitivity to commonly used medications (particularly central nervous system and cardiovascular agents), and changes in organ function including renal, hepatic, and respiratory systems (13). Kidney function changes are particularly significant, as both kidney mass and renal blood flow decline with aging. By age 80, there is approximately a 40% reduction in functional nephrons. Failure to adjust medication doses based on renal function can result in drug accumulation, toxicity, and worsening kidney function (14). Age-related changes in body composition, including alterations in muscle mass and fat distribution, lead to increased inter-individual variability in drug distribution and consequently increased ADE risk. Water-soluble drugs often achieve higher serum concentrations due to relative decreases in total body water in geriatric patients. Conversely, the higher proportion of body fat in older adults can prolong the half-life of fat-soluble drugs such as benzodiazepines (14).

#### Functional changes and frailty impact

Functional changes related to aging, collectively termed frailty, represent another significant risk factor for ADEs. Frailty is characterized by diminished strength, endurance, and reduced physiologic function that increases vulnerability to dependency and adverse health outcomes. Frailty inherently increases with advancing age and is particularly prevalent among hospitalized and nursing home residents (14). Research has shown that the simultaneous presence of falls risk and dependency in activities of daily living—surrogate markers for frailty—is associated with increased ADE risk in hospitalized geriatric patients. Interestingly, reductions in drug metabolism have been observed in frail geriatric patients compared to more robust older adults, suggesting that frailty impacts not only physical vulnerability but also pharmacological processing (7).

### Implementation failures and suboptimal outcomes

Despite the theoretical promise of SCDS, numerous implementation failures and suboptimal outcomes have been documented in the literature, providing important lessons for future deployments. A comprehensive analysis of these challenges reveals several recurring themes that healthcare organizations must proactively address.

#### Alert fatigue and override behavior

Alert fatigue represents the most extensively documented SCDS implementation challenge. A landmark study by Phansalkar et al. found that drug-drug interaction alerts had override rates ranging from 49% to 96% across different healthcare systems, with median override rates of 87% for drug-drug interaction alerts specifically (72). These high override rates effectively negate the safety benefits of alerting systems, as clinicians become desensitized to warnings and may miss clinically significant alerts embedded among numerous low-priority notifications. Research by Ancker et al. demonstrated that the number of alerts per patient encounter inversely correlated with provider attention to individual alerts, creating a dangerous cycle where increased alerting paradoxically decreases safety (73). Several failed implementations have been attributed to poorly designed alert systems that generated excessive false-positive alerts with insufficient clinical specificity. One large academic medical center reported that after implementing a comprehensive alerting system, providers received an average of 48 alerts per patient admission, with 92% classified as clinically insignificant upon retrospective review; this implementation was subsequently rolled back and redesigned with more selective alert criteria, resulting in 75% reduction in alert volume and improved provider acceptance (74).

#### Workflow disruption and workarounds

SCDS systems that fail to integrate seamlessly with clinical workflows often prompt providers to develop workarounds that compromise patient safety. Case studies have documented providers entering dummy data to bypass mandatory fields, ignoring alerts without reading them, or using shared login credentials to avoid authentication delays. A qualitative study by Koppel et al. identified 22 distinct types of workarounds used by providers to circumvent CPOE safety features, including entering orders under incorrect patient names and using copy-forward functions that propagated outdated medication lists (75). One particularly concerning failure mode involves forcing functions that are too restrictive, preventing appropriate clinical care in time-sensitive situations. Several emergency departments reported cases where mandatory pharmacist authorization requirements for specific medications caused dangerous delays in treatment, leading to adverse outcomes (76). These implementations were subsequently modified to allow emergency override provisions while maintaining pharmacist review for non-urgent situations.

#### Interoperability and data quality issues

Fragmented health information exchange represents a fundamental barrier to SCDS effectiveness. Patients receiving care across multiple healthcare systems using different EHR platforms often have incomplete medication histories, rendering drug interaction and duplicate therapy alerts unreliable. A study by Schiff et al. found that 22% of ambulatory patients had discrepancies between their EHR medication lists and actual medications taken, with 10% of discrepancies classified as potentially harmful (77). These data quality issues undermine clinician trust in SCDS recommendations and contribute to alert override behaviors. Several healthcare systems have documented failed attempts to implement comprehensive medication reconciliation processes due to time constraints, inadequate staffing, and technical limitations (78). One large integrated delivery network abandoned a mandated medication reconciliation program after finding that the time required per patient encounter increased by an average of 12 min, creating unacceptable bottlenecks in clinical workflows without demonstrable improvement in medication accuracy (79).

#### Resistance to change and training inadequacy

Provider resistance represents a significant barrier to SCDS adoption, particularly among clinicians who perceive decision support as questioning their clinical judgment or imposing unnecessary restrictions on prescribing autonomy (80). Several implementations have failed due to inadequate stakeholder engagement during planning phases, resulting in systems that did not meet clinical needs or align with established practice patterns. Training inadequacy has been implicated in multiple suboptimal implementations. One community hospital network reported that only 35% of providers felt competent using SCDS features six months after implementation despite completing required training, suggesting that initial training was insufficient for developing practical competency (81). Subsequent analysis revealed that training focused on technical navigation rather than clinical application and decision-making support, limiting provider ability to effectively utilize system capabilities.

#### Unintended consequences

Several studies have documented unintended negative consequences of CDSS implementation, including increased medication errors due to confusing interfaces, alert misinterpretation, and assumption that the computer would catch all errors (82). One systematic review by Ash et al. identified nine categories of unintended adverse consequences, including workflow disruption, system demands that alter clinical processes, and new types of errors related to technology use (83). A particularly concerning unintended consequence involves automation bias, where providers over-rely on CDSS recommendations without applying independent clinical judgment. Case reports have documented inappropriate medication continuation when CDSS failed to generate expected alerts due to system failures or data entry errors, with providers assuming that absence of alerts indicated appropriateness of therapy.

## Evidence-based interventions

### Intervention framework

**Figure 2:**
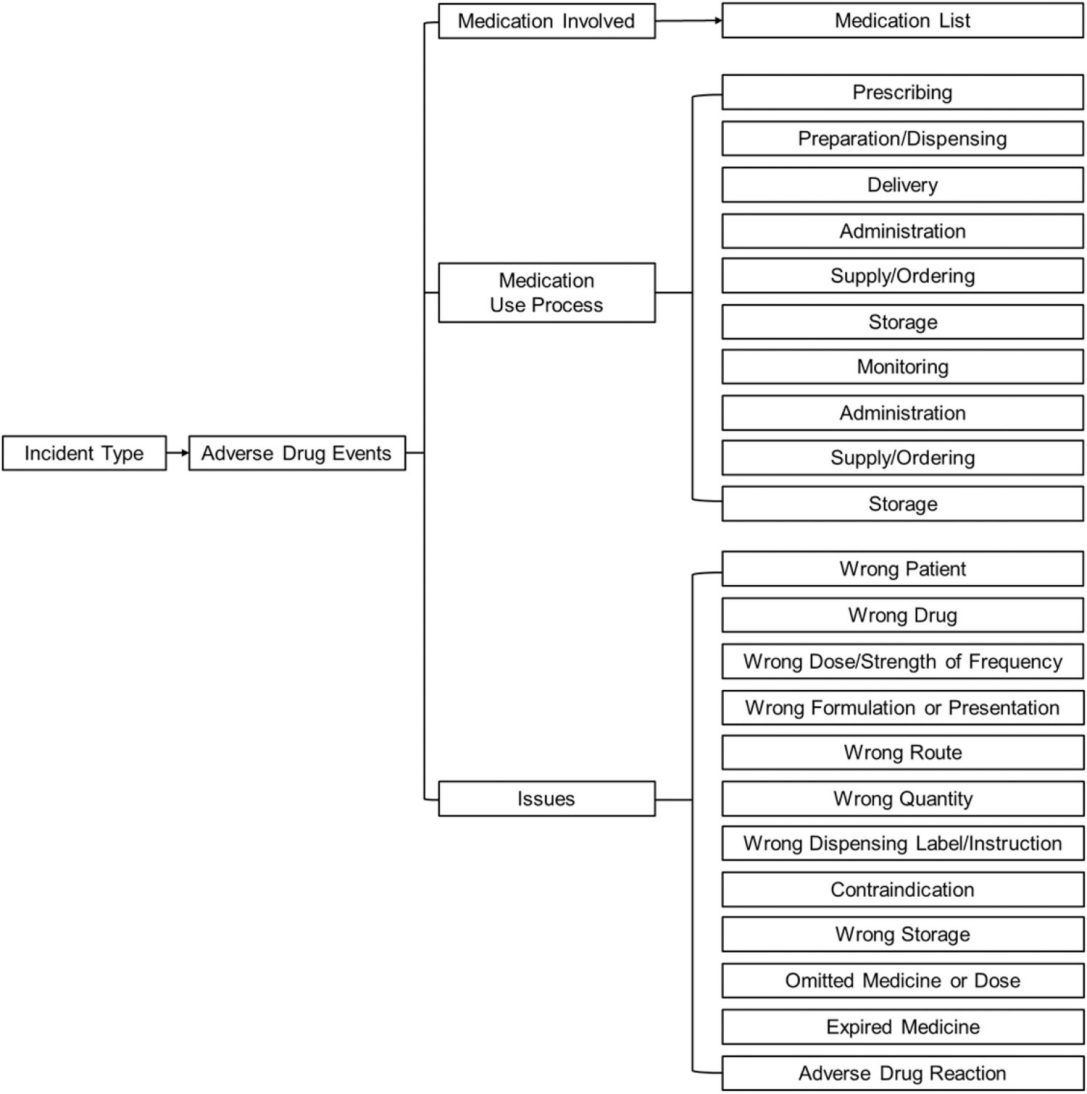
Conceptual framework of the adverse drug event.

Implementing effective interventions to reduce ADEs and improve patient safety requires a multifaceted approach that addresses the various risk factors and systemic challenges identified in healthcare delivery. We have developed a framework (Figure 2) based on the WHO Conceptual Framework for the International Classification for Patient Safety (19) to propose evidence-based recommendations for interventions utilizing SCDS. This framework illustrates how SCDS interventions can address multiple contributing factors simultaneously, including healthcare provider factors, system factors, and patient factors. Arrows indicate pathways through which interventions influence outcomes, with feedback loops demonstrating the iterative nature of continuous quality improvement in patient safety.

Table 6 illustrates SCDS intervention spectrum and effectiveness.

**Table 6:** SCDS intervention spectrum and effectiveness.

Intervention level	Description	Implementation complexity	Evidence quality
Basic Alerts			
Allergy warnings (84)	Pop-up alerts for known allergies	Low	High
Drug interaction alerts (72)	Warnings for dangerous combinations	Low-Medium	High
Duplicate therapy alerts (85)	Identification of therapeutic duplications	Low	Medium
Enhanced Alerts			
Renal/hepatic function warnings (86)	Organ function-based alerts	Medium	High
Age-appropriate dosing (87)	Geriatric-specific recommendations	Medium	High
Alternative Suggestions			
PIM replacement recommendations (88)	Safer medication alternatives	Medium–High	Medium–High
Dose optimization (89)	Patient-specific dosing recommendations	Medium–High	High
Forcing Functions			
Automatic dose adjustment (90)	System-calculated dose modifications	High	Medium
Mandatory pharmacist approval (91)	Required authorization for high-risk medications	High	High
Hard stops (92)	Prevention of dangerous prescriptions	Very High	High

### Spectrum of SCDS interventions

SCDS interventions exist along a continuum from basic alert systems to sophisticated forcing functions. At the foundational level, alert systems warn healthcare providers about patient allergies and medication interactions when prescribing new medications. These alerts serve as immediate clinical reminders, ensuring that providers consider potential adverse reactions before finalizing prescriptions (93). Basic allergy alerts notify providers when they attempt to prescribe medications to patients with documented allergies to those specific agents or drug classes. Drug interaction alerts warn about potentially dangerous combinations when providers prescribe medications that may interact with patients' current medications. These alerts can be expanded to remind providers about patients' abnormal kidney or liver function, which can significantly impact medication metabolism and safety (94).

### Evidence-based interventions

However, the effectiveness of basic alert systems remains controversial in the literature. While some studies report significant reductions in prescribing errors with basic alerts, others document high override rates (49%–96% for drug interaction alerts) that substantially diminish their impact (85, 95). This discrepancy likely reflects differences in alert specificity, clinical relevance, and integration with clinical workflows. Studies with lower override rates typically employed more selective alerting strategies, limiting alerts to high-severity interactions and incorporating clinical context into alert triggering logic (85, 96).

### Advanced SCDS capabilities

More sophisticated SCDS implementations suggest appropriate alternative medications or dosing adjustments when potentially inappropriate medications (PIMs) or doses are entered by providers (37). For example, if a provider enters a prescription for a medication contraindicated due to patient renal insufficiency, the system could recommend an alternative drug or suggest a safer dose based on calculated creatinine clearance. Advanced SCDS systems can incorporate forcing functions that automatically adjust medication doses based on renal or hepatic function. In patients with impaired kidney function, the system could automatically calculate and recommend dose adjustments for renally-cleared medications, ensuring safer medication use without requiring manual calculations by providers (95).

### Pharmacist integration and oversight

The most robust SCDS implementations may require providers to obtain pharmacist authorization before prescribing PIMs. This intervention involves pharmacist review of the prescription with either approval or alternative suggestions, adding a crucial layer of safety oversight. Pharmacists can identify less risky medications that still meet patients' therapeutic needs while minimizing ADE risk.

### Evidence for intervention effectiveness

The effectiveness of these interventions in mitigating polypharmacy, comorbidity, and physiologic change-related risks depends on their implementation strength and workflow integration. Basic alerts may address some risks but may be insufficient for complex clinical scenarios. Stronger interventions, particularly those involving forcing functions, can significantly reduce ADE risk by ensuring more consistent adherence to clinical guidelines (85). Research by Raebel et al. demonstrated significant decreases in inappropriate medication orders when provider-pharmacist communication regarding inappropriate medication prescribing was required, underscoring the value of integrated, multidisciplinary approaches that combine technological tools with human expertise (97).

### The beers criteria and educational interventions

The American Geriatrics Society Beers Criteria is a widely recognized tool that specifies potentially inappropriate medications (PIMs) for use in geriatric patients, aiming to reduce adverse clinical outcomes, hospitalizations, mortality, and health care costs associated with these medications (98). The Beers Criteria lists medications that are particularly risky for older adults due to their association with ADEs (99). This list helps mitigate risks related to the physiological and functional changes that occur with aging by identifying medications that are specifically linked to adverse events in geriatric patients. These changes include reduced organ function, altered pharmacokinetics, and increased vulnerability to drug side effects. However, the Beers Criteria also highlights a common knowledge gap among health care providers who are not geriatric specialists, as many medications deemed inappropriate for older adults may be safe for younger populations. While the Beers Criteria provides valuable guidance, it is primarily an educational tool and, as such, is inherently less effective than other interventions that actively enforce safer prescribing practices. Moreover, the Beers Criteria addresses only a fraction of the overall risk, as Beers Criteria medications are implicated in only 16.5% of geriatric ADEs (100). Despite its limitations, the Beers Criteria represents an important first step in addressing ADEs in geriatric patients by targeting providers' knowledge gaps. Table 7 demonstrates the American Geriatrics Society Beers Criteria for High-Risk Medication Categories (101).

**Table 7:** The American geriatrics society beers criteria—high-risk medication categories.

Medication category	Risk level	ADE types	Alternative considerations	Monitoring requirements
Central Nervous System				
Benzodiazepines (14)	High	Sedation, falls, cognitive impairment	Non-pharmacologic approaches, shorter-acting alternatives	Cognitive assessment, fall risk
Antipsychotics (99)	Very High	Metabolic syndrome, movement disorders	Behavioral interventions, mood stabilizers	Metabolic monitoring, movement assessment
Tricyclic antidepressants (102)	High	Anticholinergic effects, cardiac arrhythmias	SSRIs, SNRIs	Cardiac monitoring, anticholinergic burden
Cardiovascular				
Alpha-blockers (103)	Medium–High	Orthostatic hypotension, falls	ACE inhibitors, ARBs	Blood pressure monitoring, fall assessment
Anti-arrhythmics (103)	High	Pro-arrhythmia, drug interactions	Rate control, anticoagulation	Cardiac monitoring, drug levels
Endocrine				
Sulfonylureas (10)	Medium–High	Hypoglycemia	Metformin, DPP-4 inhibitors	Blood glucose monitoring
Sliding scale insulin (101)	Medium	Hypoglycemia, poor glycemic control	Basal-bolus regimens	Blood glucose monitoring
Other High-Risk Categories				
Nonsteroidal Anti-Inflammatory Drugs (NSAIDs) (104)	High	GI bleeding, renal impairment	Topical agents, acetaminophen	Renal function, GI assessment
Muscle relaxants (105)	High	Sedation, falls	Physical therapy, topical agents	Fall risk assessment

### Specialist teams and multidisciplinary approaches

Specialist teams, historically used to manage patients with specific conditions such as HIV or Clostridioides difficile infections, offer promising approaches to ADE prevention (106). These multidisciplinary teams typically include specialized nurses, clinical pharmacists, social workers, specialist consultants, transitional care coordinators, and rehabilitation therapists (98). By addressing patients' functional status and coordinating comprehensive care, these teams can significantly reduce frailty and functional changes that heighten ADE risk (107). Specialist teams focus on medication review and management, identifying and mitigating risks associated with polypharmacy, comorbidities, and age-related physiologic changes. Table 8 illustrates the specialist team interventions and outcomes.

**Table 8:** Specialist team interventions and outcomes.

Team composition	Primary focus	Measured outcomes	ADE impact	Implementation considerations
Geriatric Emergency Department Teams				
Geriatricians, specialized nurses, pharmacists, social workers (108)	Acute geriatric care, medication review	Decreased ED length of stay, reduced admissions	Moderate reduction	Requires specialized training, dedicated space
Acute Care for Elders (ACE) Units				
Interdisciplinary team with geriatric focus (109)	Inpatient geriatric care, preventing complications	Reduced length of stay, lower readmission rates	Significant reduction	High resource requirements, staff expertise
Geriatric Assessment Teams				
Geriatricians, nurses, pharmacists, therapists, social workers (110)	Holistic assessment, care planning	Improved functional status, reduced institutionalization	Significant reduction	Requires coordination across disciplines
Medication Reconciliation Teams				
Pharmacists, nurses, physicians (111)	Accurate medication histories, transition management	Reduced medication errors, improved adherence	High reduction	Technology integration, time intensive

Research demonstrates that models incorporating specialist teams result in improved outcomes, including decreased hospital admission rates, reduced dependency levels, and lower mortality rates (112). Specialist units within hospitals and emergency departments provide targeted care with goals of reducing hospital length of stay and preventing unnecessary admissions. These units foster provider expertise and have demonstrated success in decreasing both length of stay and readmission rates (113, 114).

## Future directions and innovation opportunities

### Artificial intelligence integration

The future of SCDS lies in the integration of artificial intelligence (AI) technologies to enhance predictive capabilities and personalize interventions. AI-powered systems can analyze vast amounts of patient data to identify subtle patterns that may indicate increased ADE risk, enabling proactive interventions before adverse events occur. Table 9 shows the emerging technologies in SCDS.

**Table 9:** Emerging technologies in SCDS.

Technology	Current Development stage	Potential ADE impact	Key challenges
Artificial Intelligence			
Predictive analytics (115)	Early implementation	High—proactive risk identification	Data quality, algorithm validation
Natural language processing (116)	Pilot studies	Medium—improved documentation analysis	Clinical context understanding
Machine learning algorithms (117)	Research phase	High—pattern recognition	Training data requirements
Advanced Decision Support			
Personalized medicine integration (118)	Development	Very high—individualized therapy	Genetic testing integration
Real-time monitoring integration (119)	Pilot implementation	High—continuous assessment	Sensor technology, data processing
Patient-Centered Technologies			
Mobile health applications (120)	Early deployment	Medium—patient engagement	User adoption, digital literacy
Wearable device integration (121)	Development	Medium–High—physiologic monitoring	Data integration, accuracy

### Genomic medicine and pharmacogenomics

The integration of pharmacogenomic testing into SCDS represents a significant opportunity for personalizing medication therapy and reducing ADEs (122). Understanding individual genetic variations that affect drug metabolism can guide medication selection and dosing decisions, particularly for medications with narrow therapeutic windows or known genetic variations in metabolism.

### Real-time physiological monitoring

Advanced monitoring technologies, including wearable devices and continuous physiological sensors, can provide real-time data about patient responses to medications. This information can be integrated into SCDS to provide immediate alerts about potential adverse reactions and enable rapid intervention (123).

### Patient-generated health data integration

The incorporation of patient-generated health data from mobile applications, wearable devices, and patient-reported outcome measures can enhance SCDS by providing comprehensive pictures of patient responses to medications outside clinical settings (124). This information can identify ADEs that might otherwise go undetected in traditional healthcare encounters.

### Healthcare economic impact of ADEs

The economic burden of ADEs extends far beyond immediate treatment costs, encompassing direct medical expenses, indirect costs associated with lost productivity, and long-term healthcare utilization increases. Understanding these economic implications is crucial for justifying investments in SCDS implementation. Economic analyses consistently demonstrate that investments in SCDS technology and implementation generate significant returns through ADE reduction (125).

### Value-based care integration

SCDS aligns well with value-based care models that emphasize patient outcomes and cost-effectiveness rather than volume of services. Healthcare organizations participating in value-based contracts can leverage SCDS to improve quality metrics while reducing overall costs of care (126).

### Quality metrics and performance measurement

Measuring the effectiveness of SCDS implementation requires monitoring of multiple performance indicators that reflect both process improvements and clinical outcomes. Table 10 presents the SCDS performance metrics.

**Table 10:** SCDS performance metrics.

Metric category	Specific measures	Monitoring frequency	Data sources
Clinical Outcomes			
ADE incidence rate (127)	ADEs per 1,000 patient days	Monthly	EHR, incident reports
Preventable ADE rate (127)	Preventable ADEs per 1,000 patient days	Monthly	EHR, clinical review
ADE severity distribution (128)	Mild/moderate/severe ADE percentages	Quarterly	Clinical assessment
Process Measures			
Alert response rate (129)	Percentage of alerts acted upon	Weekly	CDSS logs
Override rates (130)	Percentage of safety alerts overridden	Weekly	System analytics
Time to intervention (129)	Hours from alert to clinical action	Daily	Workflow analysis
Patient Engagement			
Portal utilization (24)	Percentage of patients using portal	Monthly	Portal analytics
Patient satisfaction (131)	Satisfaction scores with shared decision-making	Quarterly	Patient surveys
System Performance			
System uptime (132)	Percentage of time SCDS available	Continuous	IT monitoring
Response time (133)	Average system response time	Continuous	Performance monitoring

### Regulatory and legal considerations

SCDS implementation must comply with various regulatory requirements, including HIPAA privacy and security regulations, FDA guidelines for medical device software, and Joint Commission patient safety standards. Table 11 demonstrates the regulatory compliance framework for SCDS.

**Table 11:** Regulatory compliance framework for SCDS.

Regulatory domain	Key requirements	Compliance strategies	Monitoring approaches
Privacy and Security			
HIPAA compliance (134)	Protected health information security	Encryption, access controls, audit trails	Regular security assessments
Data governance (135)	Appropriate data use and sharing	Governance committees, policies	Audit processes
Clinical Decision Support			
FDA regulations (136)	Medical device software requirements	Quality management systems	Regulatory submissions
Clinical validity (137)	Evidence-based decision support rules	Clinical evidence documentation	Performance monitoring
Patient Safety			
Joint Commission standards (138)	Patient safety goals compliance	Safety committees, reporting systems	Accreditation surveys
Risk management (139)	Liability and risk mitigation	Insurance, legal review	Incident analysis

Healthcare organizations implementing SCDS must carefully consider liability implications and develop risk management strategies. This includes ensuring appropriate clinical validation of decision support rules, maintaining adequate professional liability coverage, and establishing clear protocols for system failures or unexpected outcomes (140).

## Discussion

### Overview

The review of shared clinical decision support demonstrates significant potential for reducing adverse drug events and improving patient safety outcomes. SCDS represents a paradigm shift toward patient-centered care that leverages technology to enhance clinical decision-making while empowering patients to actively participate in their healthcare decisions. The evidence clearly supports a multifaceted approach to ADE prevention that combines educational initiatives, human factors engineering, robust shared clinical decision support systems, and multidisciplinary collaborative care models. The most effective implementations integrate strong technological capabilities with human expertise and continuous quality improvement methodologies.

### Critical analysis of implementation challenges

While our review demonstrates the potential of SCDS to reduce ADEs, successful implementation faces substantial obstacles that warrant careful consideration. The gap between theoretical promise and practical implementation remains considerable across healthcare settings. Alert fatigue represents one of the most significant barriers to SCDS effectiveness, with studies indicating that clinicians override 49%–96% of drug interaction alerts, potentially missing critical safety warnings (141). This desensitization occurs when clinicians are overwhelmed by excessive, non-specific, or clinically irrelevant alerts, leading to dangerous override behaviors that undermine system effectiveness.

Interoperability challenges further complicate SCDS implementation, particularly in healthcare systems utilizing multiple electronic health record platforms or serving patients who receive care across different healthcare organizations. Incomplete medication histories due to fragmented health information exchange can result in missed drug interactions or duplicate therapy alerts, paradoxically increasing ADE risk despite SCDS deployment. The lack of standardization in clinical decision support rules across different systems creates inconsistencies in care delivery and may confuse providers practicing in multiple settings.

### Comparative effectiveness of interventions

Our analysis reveals a spectrum of intervention effectiveness, with forcing functions and mandatory pharmacist review demonstrating superior outcomes compared to basic alert systems. However, these more robust interventions require greater implementation complexity, resource investment, and workflow integration. Healthcare organizations must balance intervention strength against feasibility and user acceptance, as overly restrictive systems may prompt workaround behaviors that compromise patient safety. Translating these models to community hospitals and rural healthcare settings presents significant challenges related to staffing, expertise availability, and financial sustainability (142). Further research is needed to develop scalable implementation models that maintain effectiveness while accommodating resource constraints in diverse healthcare settings.

### Limitations

This narrative review has several limitations that should be acknowledged. First, our search strategy was not exhaustive, and we may have missed relevant studies, particularly those published in non-English languages or in specialty journals outside our search parameters. Second, the included studies may limit generalizability to healthcare systems with different organizational structures, regulatory environments, and cultural approaches to clinical decision-making. Third, the heterogeneity of SCDS interventions described in the literature made direct comparisons challenging, and we were unable to conduct meta-analyses of intervention effectiveness.

### Recommendations for future research

Future research should prioritize comparative effectiveness studies that rigorously evaluate different SCDS intervention strategies across diverse healthcare settings, patient populations, and geographic regions. Head-to-head comparisons of basic alerts vs. forcing functions, different pharmacist integration models, and various specialist team configurations would inform evidence-based implementation decisions and help healthcare organizations select optimal strategies for their specific contexts.

Implementation science research employing established frameworks should examine barriers and facilitators to SCDS adoption, strategies for overcoming alert fatigue, and methods for sustaining system effectiveness over time (143). Mixed-methods approaches combining quantitative outcomes with qualitative exploration of provider and patient experiences would provide valuable insights into the human factors influencing implementation success and identify modifiable factors that can be addressed through targeted interventions (144).

Comprehensive cost-effectiveness analyses comparing different SCDS implementation strategies would support resource allocation decisions and business case development (145). These economic evaluations should include long-term return on investment calculations, assessment of indirect benefits such as improved patient satisfaction and reduced liability exposure, and consideration of implementation and maintenance costs across diverse healthcare settings with varying resource availability (146).

As AI technologies mature, rigorous evaluation of AI-enhanced SCDS systems through randomized controlled trials and pragmatic clinical studies should assess both effectiveness and potential unintended consequences (147). Research should examine AI systems' ability to predict ADE risk, personalize interventions based on individual patient characteristics, and improve upon traditional rule-based decision support while carefully monitoring for algorithmic bias, inappropriate automation reliance, and other novel safety concerns (148).

Finally, longitudinal research examining sustained effects of SCDS implementation on clinical outcomes, healthcare costs, and patient satisfaction over extended time periods would demonstrate durability of benefits and identify factors associated with sustained effectiveness. These studies should investigate whether initial improvements are maintained over time, how systems adapt to changing clinical guidelines and technologies, and what organizational characteristics predict long-term implementation success.

## Conclusion

The transformation of healthcare delivery through SCDS implementation represents both a significant opportunity and a substantial challenge. Success requires sustained commitment from healthcare organizations, continued investment in technology infrastructure, and ongoing dedication to patient safety improvement. The potential benefits—reduced patient harm, improved healthcare quality, decreased costs, and enhanced patient satisfaction—justify the substantial effort required for successful implementation. As healthcare continues to evolve toward value-based care models, SCDS will play an increasingly important role in achieving the triple aim of better patient experience, improved population health, and reduced per capita healthcare costs. The integration of emerging technologies, including AI, genomic medicine, and real-time physiological monitoring, will continue to enhance SCDS capabilities and expand its impact on patient safety. Healthcare organizations that invest in SCDS implementation today will be well-positioned to realize these future benefits and lead the transformation toward safer, more effective healthcare delivery. The ultimate goal of SCDS implementation extends beyond simply reducing adverse drug events to creating a healthcare system that consistently delivers the right care, to the right patient, at the right time, in the right setting, with optimal outcomes and minimal risk. This vision requires continued innovation, sustained commitment, and collaborative effort from all healthcare stakeholders, but the potential benefits for patients, providers, and healthcare systems justify this substantial undertaking.
